# The Nucleoside Analogue NITD008, Alone or Combined With Ribavirin, Potently Reduces Hepatitis E Virus Infection in Humanised Mice

**DOI:** 10.1111/liv.70955

**Published:** 2026-11-11

**Authors:** Annika Volmari, Florian Hinte, Tassilo Volz, Junfei Chen, Lena Allweiss, Marc Lütgehetmann, Maura Dandri

**Affiliations:** ^1^ I. Department of Internal Medicine University Medical Center Hamburg‐Eppendorf Hamburg Germany; ^2^ German Center for Infection Research (DZIF) Hamburg Germany; ^3^ Department of Medical Microbiology, Virology and Hygiene University Medical Center Hamburg‐Eppendorf Hamburg Germany

**Keywords:** antiviral therapy, hepatitis E, HEV, humanised mice, viral polymerase inhibitors

## Abstract

**Background and Aims:**

Hepatitis E virus (HEV) infection is becoming increasingly clinically relevant worldwide, but no approved antiviral therapy currently exists. Ribavirin (RBV) is used off‐label for immunosuppressed patients with chronic hepatitis E, although it is effective in only 80% of patients and linked to side effects. The nucleoside analogue NITD008 was shown to be effective against a broad range of RNA viruses. We aimed to evaluate the efficacy of NITD008, alone or in combination with RBV, in humanised mice stably infected with HEV.

**Methods:**

Mice received either NITD008 alone (20 mg/Kg/day) by oral gavage for up to 2 weeks, or 5 mg/Kg NITD008 alone or in combination with RBV (50 mg/Kg). Viral changes were determined in faeces, blood and intrahepatically.

**Results:**

NITD008 efficiently reduced HEV loads to undetectable levels in serum and by > 4log_10_ in faeces within 1 week. Intrahepatic analysis showed a ∆log_10_: 2.5 reduction in HEV RNA levels in treated mice compared to controls after 2 weeks. Imaging showed fewer HEV‐RNA‐positive human hepatocytes in treated mice. NITD008 did not increase serum markers of hepatocyte death. Combination therapy induced stronger suppression of HEV RNA in faeces (∆log_10_: 5.02) than RBV monotherapy (∆log_10_: 1.08), despite lower NITD008 doses.

**Conclusions:**

This study demonstrates the non‐toxicity of NITD008 towards human hepatocytes and more potent antiviral activity compared to RBV in HEV‐infected mice. Combination regimens with RBV showed strong additive activity. The study suggests that HEV clearance may be achieved in immunosuppressed patients by using and further developing this class of direct antiviral drugs.

## Key Points

- The therapeutic potential of the adenosine analogue NITD008 against HEV was tested in a liver‐chimeric humanised mouse model.
- NITD008 showed high anti‐viral potency against HEV in vivo, alone and in combination with Ribavirin.
- No liver toxicity was detected following the treatment with NITD008 in vivo.
- The study highlights the therapeutic potential of this class of viral polymerase inhibitors to treat HEV infections.

**Abbreviations**
ALT: alanine aminotransferase
CK‐18: cytokeratin‐18
GAPDH: glyceraldehyde‐3‐phosphate dehydrogenase
HCV: Hepatitis C Virus
HEV: Hepatitis E Virus
ISH: in situ hybridisation
LoQ: lower limit of quantification
ORF: open reading frame
Peg‐IFNα: pegylated Interferon alpha
PHH: primary human hepatocytes
RBV: Ribavirin
RdRp: RNA‐dependent RNA polymerase
RPL30: ribosomal protein L30
RT‐qPCR: reverse transcription quantitative polymerase chain reaction
uPA: urokinase plasminogen activator
USG: uPA/SCID/beige/IL2RG^−/−^

## 1 Introduction

Hepatitis E virus (HEV, Paslahepevirus balayani) infection poses a significant global health threat, particularly in developing countries where outbreaks are common and in industrialised nations where zoonotic transmission occurs. Around 20 million HEV infections have been estimated to occur annually worldwide, resulting in an estimated loss of 5.4% of global disability‐adjusted life years (DALYs) related to acute hepatitis. While HEV infection is often self‐limiting in healthy individuals, it can lead to severe and potentially fatal outcomes in specific populations. HEV infection is associated with high morbidity and mortality rates (up to 25%) in pregnant women [1] and is linked with chronic progression with rapid cirrhosis development in patients with pre‐existing liver damage and in immunocompromised patients (e.g., organ transplant recipients, individuals with haematologic malignancies and those living with HIV). Chronic hepatitis E, which has been reported for HEV Genotypes 3, 4 and 7 [2, 3], can lead to acute‐on‐chronic liver failure [4] and it is associated with severe extrahepatic manifestations [5].

HEV is a positive‐sense, single‐stranded RNA virus with eight known genotypes. Genotypes 1 and 2 are restricted to humans and are transmitted primarily via the faecal‐oral route via contaminated drinking water. These genotypes predominate in developing countries: Genotype 1 is present in India, parts of China and North Africa and Genotype 2 in Mexico and West Africa. Genotypes 3 and 4 are zoonotic and are generally transmitted to humans through the consumption of inadequately cooked pork and venison. Genotype 3 is predominant in Europe, Australia, North America and Japan, whereas Genotype 4 infections occur primarily in China. Genotypes 5–8 (wild boar, camels) play only a minor role in human medicine [6]. The HEV genome is organised into three open reading frames (ORFs). ORF1 encodes the enzymes required for genomic replication, including the RNA‐dependent RNA polymerase (RdRp), which synthesises antisense and sense RNA transcripts using the respective full‐length sense and antisense viral RNA templates. Shorter subgenomic RNAs are used to translate the ORF2 (encoding the capsid protein) and ORF3 (encoding the viroporin protein).

For immunosuppressed people with chronic hepatitis E, there is no HEV‐specific antiviral therapy approved. Ribavirin (RBV) is the primary off‐label treatment used in routine clinical practice [7]. Although RBV is effective in about 80% of patients [8], it is contraindicated in some patients due to the risk of haemolytic anaemia and its broad range of side effects. Moreover, viral relapse after prolonged treatment has been associated with the emergence of HEV variants within the viral population that can enhance replication efficacy, but their emergence does not necessarily confer resistance to treatment [9]. Some patients who show no response to RBV can be considered for treatment with peg‐IFNα. However, peg‐IFNα is contraindicated in transplant recipients and significant side effects limit its clinical utility. Thus, there is an urgent need to screen and develop targeted, effective and safe antiviral compounds against HEV [10].

Nucleoside analogues are a powerful class of antiviral drugs that have been successfully used to treat a variety of viral infections caused by RNA or DNA viruses [11]. NITD008 is an adenosine nucleoside analogue that acts as an inhibitor of viral RNA‐dependent RNA polymerases (RdRp) through RNA chain synthesis termination. The antiviral compound was identified in a large‐scale screen for compounds active against the Dengue virus [12]. NITD008 showed good in vivo pharmacokinetic properties and is available for oral administration. In vitro studies also demonstrated inhibition of a broad range of RNA viruses, such as tick‐borne flaviviruses, West Nile virus, Zika virus, Hepatitis C virus and Enterovirus 71 [12, 13, 14, 15, 16, 17]. Most importantly, inhibition of HEV in hepatoma cell lines and, more recently, in neuronal cells has been observed [18, 19]. Although no adverse effects were determined in rats orally dosed with NITD008 at 50 mg/Kg daily, signs of toxicity have been described after more extended treatment periods and high concentrations in dogs [12]. Nevertheless, the antiviral potency of NITD008 against HEV in vivo and potential toxicity in primary human hepatocytes (PHH) were not assessed.

The study aimed to evaluate the antiviral potency of NITD008, as a prototype adenosine nucleoside analogue, alone and in combination with RBV, against hepatitis E using stably infected immunodeficient mice harbouring livers reconstituted with PHH [20]. Using an in vivo model in which HEV stably replicates in PHH, the natural target of HEV infection, we show the strong antiviral efficacy of NITD008, used alone or in combination with RBV, thereby demonstrating the concept that this class of nucleoside inhibitors could be further developed for the treatment of hepatitis E.

## 2 Materials and Methods

### 2.1 Generation of HEV‐Infected Humanised Mice

Homozygous USG (uPA/SCID/beige/IL2RG^−/−^) mice were housed and maintained under specific pathogen‐free conditions in accordance with institutional guidelines and authorised protocols. All procedures were approved by the ethical committee of the city and state of Hamburg and were in accordance with the 3R principles. Generation of human liver chimeric mice was performed as described previously [20]. Briefly, 1 × 10^6^ thawed human hepatocytes were injected intrasplenically into 4 weeks‐old USG mice anaesthetised with isoflurane. Levels of human chimerism were determined 8 weeks after transplantation by measuring human serum albumin in mouse serum (Human Albumin ELISA kit; ICL; https://www.ScienceDirect.com/topics/medicine‐and‐dentistry/human‐albumin). For this study, mice previously infected with HEV‐Gt1 served as donor animals to infect humanised mice through the faecal‐oral route (co‐housing) [20]. Infection establishment was monitored by measuring HEV RNA in faeces and blood [21].

### 2.2 Antiviral Treatments

Mice received the polymerase inhibitor NITD008 as monotherapy at a concentration of either 20 mg/Kg body weight or 5 mg/Kg, whereas control mice received the vehicle buffer (6 N HCl, 1 N NaOH, 100 mM citrate buffer (pH 3.5) in a mixture of 0.9:2.5:96.6) via daily oral gavage [17]. RBV (50 mg/Kg) was administered daily alone or in combination with 5 mg/Kg NITD008. Faeces and blood were collected at the specified times and each animal's weight was monitored. Liver specimens removed at the time of sacrifice were frozen in 2‐methylbutane for histological and molecular analyses.

### 2.3 RNA Extraction, Reverse Transcription Quantitative Polymerase Chain Reaction (RT‐qPCR)

Viral RNA was extracted from serum samples (5 μL) using the QiAmp MiniElute Virus Spin Kit (Qiagen). HEV RNA copies were quantified by one‐step reverse transcription quantitative PCR (RT‐qPCR) (ViiA 7 Real‐Time PCR System, Life Technologies, Carlsbad, CA) with HEV‐specific primers and probes (JVHEVF 5′GGTGGTTTCTGGGGTGAC; JVHEVR 5′AGGGGTTGGTTGGATGAA, TaqMan minor groove binding probe JVHEVPh 5′FAM‐TGATTCTCAGCCCTTCGC‐MGB). Cycling conditions were as follows: initiation step at 50°C for 5 min, 40 cycles of amplification with each cycle consisting of 95°C for 3 s and 60°C for 30 s. Serial dilutions of an HEV‐containing plasmid were used as a standard for quantification. Faeces samples (~7‐10 mg) from treated and control mice were dissolved and shredded in 1 mL Cobas PCR Media with ceramic beads. This solution was further processed and analysed as recommended by the manufacturers.

To quantify intrahepatic HEV RNA levels, total RNA was extracted from mouse liver samples as previously described [20] using the RNeasy RNA purification kit (Qiagen). Shortly, on dry ice, ~20 mg of liver was cut from frozen liver tissue specimens and transferred into a 1.5 mL tube. The RNA extraction was done according to the manufacturer's protocol. This procedure was done in triplicate. HEV RNA quantification was performed using one‐step RT‐qPCR as described above. The expression of the human housekeeping genes GAPDH (TaqMan Gene Expression assay ID: Hs99999905_m1) and RPL30 (TaqMan Gene Expression assay ID: Hs00265497_m1) was used for normalisation.

### 2.4 Liver Biomarkers

Levels of alanine aminotransferase (ALT) were determined in mouse serum samples using Fuji Dri‐Chem N × 500 (Fujifilm). Human cytokeratin‐18 (CK‐18) levels in mouse serum were measured using an enzyme‐linked immunosorbent (ELISA) assay (ab227896, abcam).

### 2.5 Immunofluorescence

HEV proteins were visualised on cryo‐preserved, acetone‐fixed liver sections by using HEV ORF2‐ (MAB8002, Millipore) and ORF3‐specific antibodies (bs‐0212R, Bioss). Human hepatocytes were co‐stained with anti‐LaminB1 (HS‐404017, Synaptic Systems) antibodies, which do not stain murine hepatocytes. Specific signals were visualised using Alexa Fluor 555‐ or 647‐labelled secondary antibodies (Invitrogen) or the TSA Fluorescin System (Perkin Elmer) to amplify HEV‐derived signals. Nuclear staining was achieved with Hoechst 33258 (Invitrogen). Stained sections were analysed by fluorescence microscopy (BZ‐9000, Keyence, Japan).

### 2.6 RNA In Situ Hybridisation (RNAscope)

RNA in situ hybridisation was performed on cryo‐preserved, paraformaldehyde‐fixed liver sections using the RNAscope Fluorescent Multiplex Kit (ACD) according to the manufacturer's instructions. Shortly, liver sections were dehydrated and pre‐treated with Protease IV (ACD) for 30 min. Samples were incubated with ORF2/3‐specific target probes (HEV‐Gt1, RNAscope assay number 442751) for 2 h at 40°C (HybEZ oven, ACD). DAPI staining was performed to visualise nuclei. Stained sections were analysed by fluorescence microscopy (BZ‐9000, Keyence) using a 60×/1.40 NA oil objective. Merged *z*‐stack images were prepared using the same settings for all groups.

### 2.7 Targeted Amplicon Next‐Generation Sequencing of the HEV RdRp Region

Stool was homogenised in 1 mL of molecular‐grade water using a Precellys 24 homogeniser (Bertin Technologies, Montigny‐le‐Bretonneux, France) to yield a 10% (w/v) suspension and clarified by centrifugation. Nucleic acids were extracted from 200 μL of the clarified supernatant on a MagNA Pure 96 system (Roche Diagnostics, Mannheim, Germany). Virus‐specific amplification was performed with the SuperScript IV One‐Step RT‐PCR System (Thermo Fisher Scientific, Waltham, MA, USA) according to the manufacturer's instructions, including the optional DNase digestion step. Briefly, 10 μL of the digested RNA eluate was used in a 50 μL primer‐specific RT‐PCR (800 nM final primer concentration) run on a ProFlex PCR System (Thermo Fisher Scientific). The following primers were used: HEV‐298_F (5′‐GATCCCAGCCATTGACTTCGAA‐3′) and HEV‐299_R (5′‐TAGCACACTCAAGGCCCAAG‐3′) for fragment 1 [22] and HEV1e_64_F (5′‐CGCTGCGAGGACATCCAT‐3′) and HEV1e_1153_R (5′‐CCAGCTGTGGCAGGTCTAC‐3′) for fragment 2.RT and cycling conditions were as follows: RT at 55°C for 10 min, followed by 5 cycles of 98°C for 30 s, 45°C for 30 s and 72°C for 2 min and 31 cycles of 98°C for 30 s, 55°C for 30 s and 72°C for 2 min. Amplicons were pooled and purified using VAHTS DNA Clean Beads (Vazyme, Nanjing, China). A total of 50–100 ng of DNA was used for Illumina library preparation with the DeepChek NGS Library Preparation Assay and DeepChek UDI Adapters (96 tests) (ABL Diagnostics S.A., Woippy, France). Libraries were normalised to 80 pM, pooled and sequenced on an Illumina iSeq 100 system (Illumina, San Diego, CA, USA) using 2 × 150 bp paired‐end chemistry (iSeq 100 i1 Reagent v2 Kit, Illumina). FASTQ files were analysed using Geneious Prime software version 2026.1.2 (Geneious, Auckland, New Zealand). FASQ reads were mapped individually to a closely related HEV genotype 1e reference sequence (GenBank accession no. OQ077986) and consensus sequences were generated at a detection limit of minorities of 5% minimum identity using ambiguity codes.

### 2.8 Data Analysis and Statistics

Graphs and statistical analyses were performed using GraphPad Prism V11.0.2. Data were reported either as individual values per mouse or as median and range. For comparisons of two groups, the Mann–Whitney test was performed and for comparisons between three groups, the Kruskal–Wallis test and Dunn's multiple comparisons test were performed.

## 3 Results

### 3.1 Reduction of HEV Loads in Serum and Faeces by NITD008 Monotherapy

Mice displaying high levels of human liver chimerism were infected with HEV (Gt1) using the co‐housing faecal‐oral route to establish chronic viral infection [21]. As depicted in the experimental design (Figure 1A), daily NITD008 administration (20 mg/Kg) was initiated 6–8 weeks after starting the co‐housing, when mice had reached stable HEV infection levels (median HEV RNA viraemia 1.26 × 10^6^IU/ml).

**FIGURE 1:**
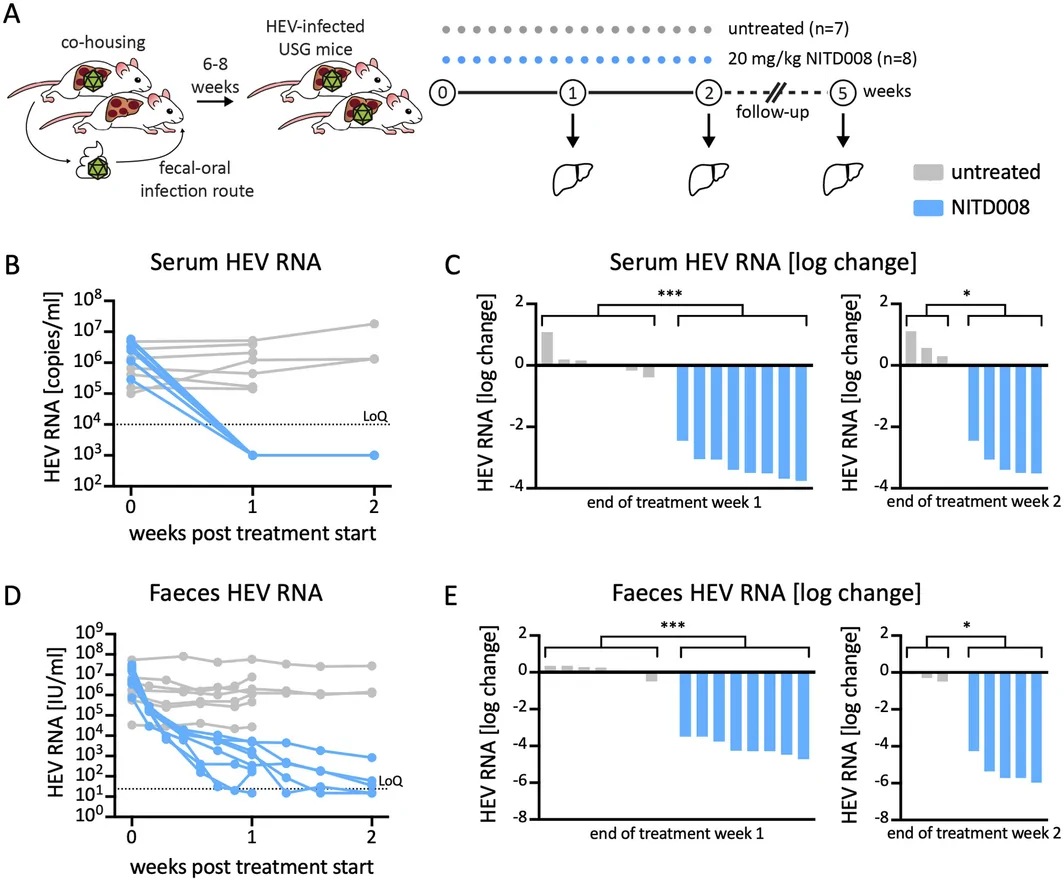
Schematic representation of experimental design and HEV RNA levels in faeces and serum. Stably infected mice received NITD008 via oral gavage daily for 1 (*n* = 3) or up to 2 weeks (*n* = 5), while a control group (*n* = 4 + 3) was left untreated. Three mice were sacrificed after 1 and 2 weeks of treatment, respectively, to assess intrahepatic changes, while 2 mice treated for 2 weeks were followed for an additional 3 weeks after treatment stop to assess rebound kinetics (A). Serum viral titres across the course of the experiment (B) and waterfall plots showing individual Log_10_ changes from baseline of HEV RNA at the end of treatment (C) determined by qPCR. Faecal viral titres across the course of the experiment (D) and waterfall plots showing individual Log_10_ changes from baseline of HEV RNA at the end of treatment (E) determined by using the Cobas system. Statistical analysis comparing the individual Log10 changes at end of treatment to baseline between the groups was performed using the Mann–Whitney test. *p < 0.05, ***p < 0.001.

Treatment with NITD008 reduced HEV RNA levels in serum (Figure 1B,C) by Δlog_10_: 3.20 (*p* = 0.0003) and by Δlog_10_: 3.40 after 2 weeks compared to baseline levels (*p* = 0.0357), with mice falling below the limit of quantification already after 1 week. Relative changes to baseline of HEV RNA in faeces (Figure 1D,E) showed a median ∆log_10_: 4.26 reduction after 1 week (*p* = 0.0002) and a ∆log_10_: 5.72 reduction after 2 weeks of NITD008 administration (*p* = 0.0357), while levels in control mice did not change substantially. Treatment was stopped in 2 animals, which were monitored for an additional 3 weeks to assess a potential viral rebound. As shown in Figure S1A,B, HEV RNA levels slowly rebounded in faeces and serum, although HEV RNA initially remained below detection in the blood of one animal (Figure S1B). To screen for potential treatment‐induced variants, the viral RdRp was analysed by NGS amplicon sequencing. HEV RNA eluates from four stool samples from three mice were used: one untreated animal that was sampled after infection establishment (baseline) and at the end of the experiment, as well as stool samples from two NITD008‐treated mice that expectedly rebounded after treatment cessation (follow‐up). Samples were analysed alongside the virus stock used for infection. For sensitive detection of viral variants, an amplicon sequencing protocol based on two overlapping fragments covering the complete RdRp domain (aa 1207–1693) [23] was established. Across the entire RdRp region, only one single nucleotide substitution resulting in an amino acid exchange was identified in one mouse that was followed‐up: a lysine (K)‐to‐arginine (R) substitution at position 1582 (K1582R; reference sequence OQ077986; Figure S2). This substitution is located in motif D of the palm domain in the critical K residue ‘GLKLKVDYRPI’ and was detected exclusively in one of the two treated animals once rebound had occurred, where it was present in > 99% of reads. It was not detected in other samples (< 1% reads).

### 3.2 No Signs of Liver Toxicity in Mice Receiving NITD008

To estimate whether 2 weeks of NITD008 treatment may cause toxicity in mice and, in particular, in PHH, animal body weight, levels of transaminases and human CK18 in serum, which serves as a human‐specific biomarker for drug‐induced liver injury, were monitored throughout the experiment. Levels of transaminases in serum, which reflect potential damage occurring in the liver, regardless of the species of the hepatocytes, remained unchanged throughout the observation time (Figure 2A). Weekly measurements of serum CK18 also failed to show an increase of this human‐specific biomarker following NITD008 treatment (Figure 2B). As depicted in Figure 2C, body weight also did not substantially change in the HEV‐infected animals, neither in the untreated controls nor in the NITD008‐treated animals. Altogether, these analyses demonstrated the good tolerability and lack of hepatocyte toxicity of NITD008 when administered for 2 weeks at 20 mg/Kg.

**FIGURE 2:**
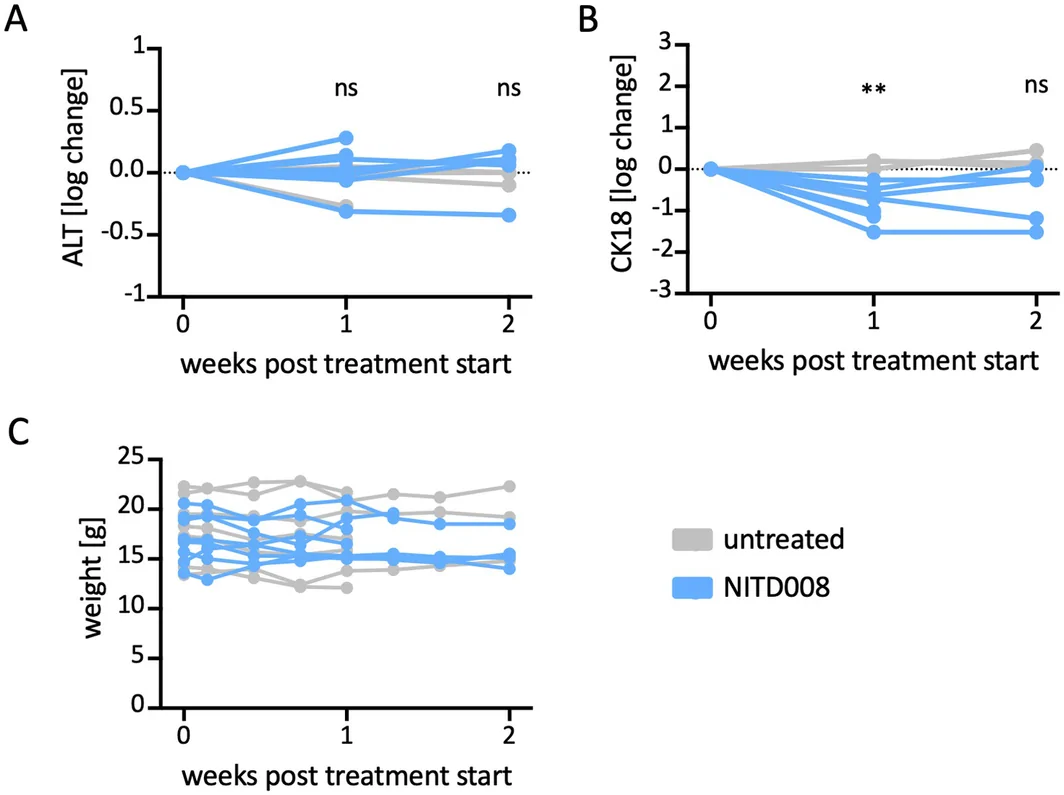
Serum hepatocyte cell death markers. Potential signs of liver toxicity following treatment of HEV‐infected mice with NITD008 for either 1 week (*n* = 3) or 2 weeks (*n* = 5) were determined by monitoring ALT (A) and human CK18 (B) in the serum compared to the untreated control group (*n* = 4 + 3). The weight of the mice was comparable throughout the experiment in both the control and treated groups (C). Statistical analysis comparing the individual Log_10_ changes after 1 and 2 weeks to baseline between the groups was performed using the Mann–Whitney test. **p < 0.01, ns = not significant (*p* > 0.05).

### 3.3 Intrahepatic HEV Changes Induced by NITD008 Monotherapy

To determine whether the reduction of HEV RNA in blood and faeces reflected the decrease of HEV infection in the liver, we assessed HEV RNA content in liver specimens of treated and control mice. Intrahepatic HEV levels declined 1.8log_10_ after 1 week and 2.7log_10_ after 2 weeks compared to control animals (*p* = 0.0022, Figure 3A). Single‐cell analysis using RNA in situ hybridisation (ISH) revealed that most human hepatocytes (positive for SERPINA1 RNA expression) were also HEV RNA positive in untreated animals. In contrast, HEV RNA signals were barely detectable in mice that received NITD008 (Figure 3B). Immunofluorescence staining of liver specimens performed using antibodies specifically recognising HEV ORF2 or ORF3 revealed far less and weaker ORF2 and ORF3 staining in mice that received NITD008 even for only 1 week, compared to the levels of HEV proteins detected in the livers of control animals (Figure 3C). Yet, these intrahepatic analyses also revealed that 2 weeks of treatment were not sufficient to achieve complete HEV clearance, thus providing a rationale for the viral rebound observed after treatment cessation in these severely immunocompromised mice.

**FIGURE 3:**
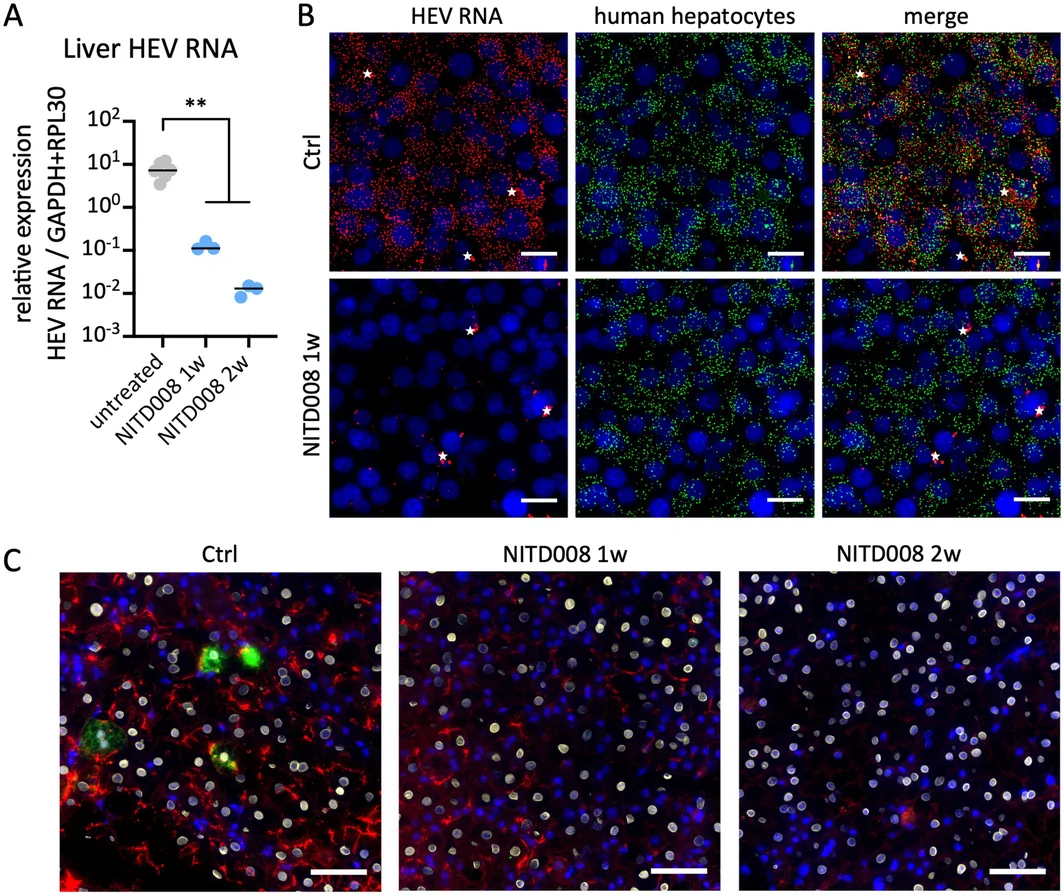
Intrahepatic HEV RNA levels and ORF2/ORF3 immunofluorescence. Relative expression of HEV RNA normalised to the human housekeeping genes GAPDH and RPL30. From every mouse, three distinct liver pieces were analysed and the mean values are shown as individual dots. Grey dots represent control mice and blue dots represent mice treated with the antiviral drug NITD008 for 1 or 2 weeks Statistical analysis comparing intrahepatic HEV RNA levels between the treated groups and untreated group was performed using Mann–Whitney test. **p < 0.01 (A). RNA in situ hybridisation (RNAscope) using HEV Gt1 probes (red) in liver sections of a representative control (top panel) and NITD008‐treated (1 week) animal (bottom panel). SERPINA1 was used for hybridisation of human RNA (green) to identify human hepatocytes in the mouse livers. Asterisks indicate autofluorescent cellular debris, which is not considered a positive staining. Scale bar represents 20 μm (B). Immunofluorescence staining of HEV proteins ORF2 (green) and ORF3 (red), respectively, in control mice and in animals treated with NITD008 for 1 week or 2 weeks. Human hepatocytes were identified with LaminB1 staining (white) and nuclei were stained with DAPI. Scale bar represents 100 μm (C).

### 3.4 Antiviral Efficacy of Lower Doses of NITD008 Used Alone or in Combination With Ribavirin

Combination therapy can significantly increase treatment efficacy and may reduce drug concentrations or shorten therapy by targeting different stages of the viral life cycle or by synergistically enhancing antiviral activity. To investigate the antiviral activity of NITD008 in comparison to RBV, both by using the drug at lower concentration and in combination with RBV, stably HEV‐infected mice (median HEV RNA viraemia 1.3 × 10^6^ IU/ml) received either RBV (50 mg/Kg) or an intentionally reduced dose of NITD008 (5 mg/Kg instead of 20 mg/Kg), whereas an additional group of mice received the two agents in combination (NITD008 5 mg/Kg and RBV 50 mg/Kg for 11 days) (Figure 4A).

**FIGURE 4:**
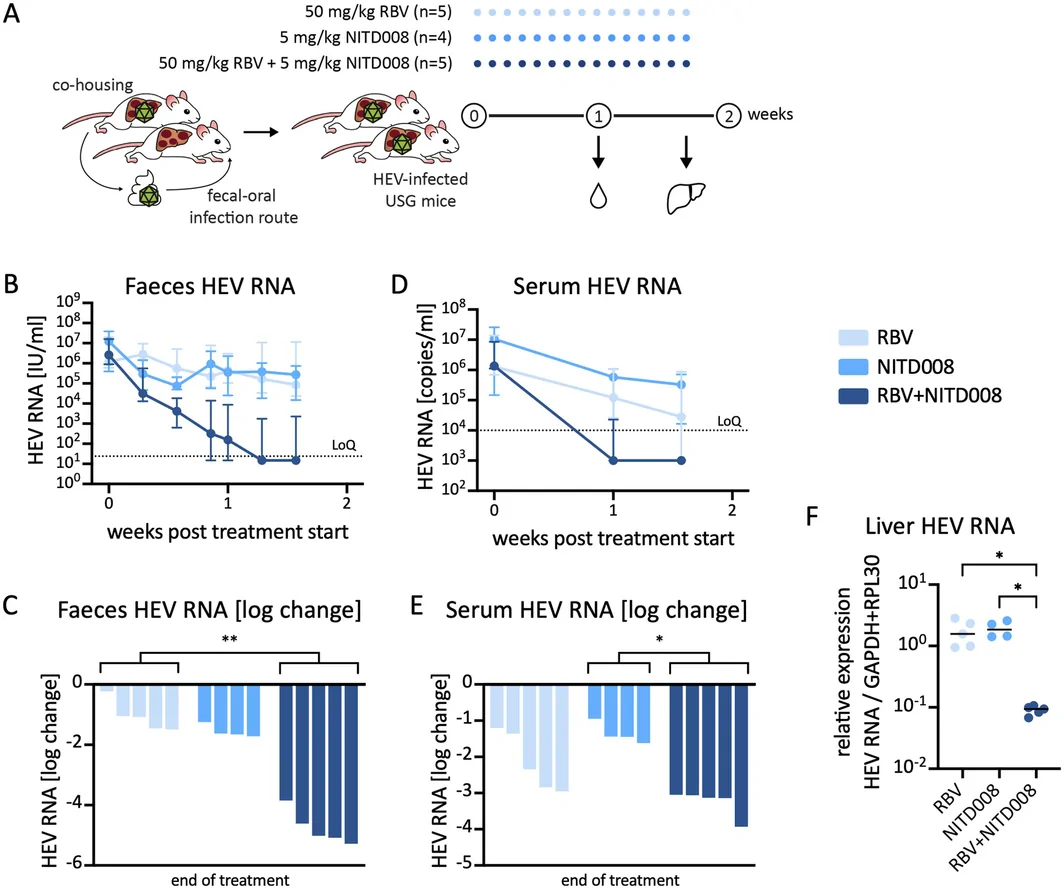
Schematic representation of experimental design and HEV RNA levels in faeces, serum and liver after combination treatment. Stably HEV‐infected mice were treated with Ribavirin (50 mg/Kg, *n* = 5), a lower dose of NITD008 (5 mg/Kg, *n* = 4), or the combination of RBV (50 mg/Kg) and NITD008 (5 mg/Kg) (*n* = 5) (A). Faecal viral titres across the course of the experiment (B) and waterfall plots showing individual Log_10_ changes from baseline of HEV RNA at the end of treatment (C) determined by using the Cobas system. Serum viral titres across the course of the experiment (D) and waterfall plots showing individual Log_10_ changes from baseline of HEV RNA at the end of treatment (E) determined by qPCR. Total HEV RNA values are shown as median and range. Intrahepatic HEV RNA levels were determined as relative expression to human housekeepers GAPDH and RPL30 (F). Statistical analysis comparing the individual Log_10_ changes at end of treatment to baseline between the groups, as well as comparing viral titres in the livers of each group was performed using the Kruskal–Wallis test, followed by Dunn's multiple comparisons test. *p < 0.05, **p < 0.01.

As shown in Figure 4B,C and in Figure S3A, treatment with RBV induced a median 1.1log_10_ reduction of viral RNA in faeces after 11 days of administration, whereas reduced amounts of NITD008 (25% compared to the previous experimental setting) led to 1.7log_10_ reduction of HEV RNA in the faeces. Remarkably, only 11 days of combination treatment elicited a rapid and strong median reduction in faecal HEV RNA load (∆log_10_: −5.0, *p* = 0.0035 compared to RBV, *p* = 0.2244 compared to NITD008) relative to baseline levels in the same animals. HEV RNA in serum was reduced in both monotherapy groups (RBV: ∆log_10_: 2.34; NITD008: ∆log_10_: 1.45), but to a greater extent in the combination therapy arm (∆log_10_: 3.13, *p* = 0.0573 compared to RBV, *p* = 0.0131 compared to NITD008) (Figure 4D,E and Figure S3B).

In the liver, RBV monotherapy and the reduced dose of NITD008 yielded comparable lower HEV RNA levels (compare also with Figure 3A), whereas the combination treatment achieved a substantially more profound reduction of HEV RNA loads (∆1.25log_10_, *p* = 0.0305 compared to RBV, *p* = 0.0293 compared to NITD008) compared to the single treatments (Figure 4D). Together, this data suggests a strong additive antiviral effect of RBV and NITD008 (20 mg/Kg/day) against HEV in vivo.

## 4 Discussion

HEV is a major aetiological factor for both acute and chronic viral hepatitis worldwide, resulting in significant morbidity and mortality [24]. Despite imposing a substantial burden on health systems worldwide, HEV is a largely understudied virus. In line with the World Health Organisation's vision of substantially reducing viral hepatitis by 2030, urgent attention must also be given to promoting research on hepatitis E and to developing treatments that effectively target HEV infection.

Antiviral development has been hampered by limitations in recapitulating the HEV life cycle in cell culture systems and by the limited availability of in vivo infection models. Human liver chimeric mice are typically generated by repopulating mouse livers with PHH. These immunodeficient animal models offer an invaluable preclinical platform for evaluating the efficacy and mode of action of both approved and novel drug candidates against human viral hepatitis [25], including against HEV [20, 21, 26]. These mice can be infected with either cell culture‐derived [27] or HEV isolates of genotype 1 or 3 derived from patients [20]. As previously reported [20, 21], transmission of HEV infection via the oral route can be effectively achieved by cohousing naïve animals with HEV‐infected mice. The model can be used either to assess strategies aiming at preventing HEV infection [21] or to establish stable HEV infection. Since human liver chimeric mice exhibit human‐like patterns of viral replication, chronic faecal viral shedding and viraemia development [20, 28], they enable in vivo investigation of antiviral compounds. Using this model, RBV was shown to cause a marked, dose‐dependent reduction in HEV viral load [20, 27]. Although the model does not allow for studying HEV‐mediated immunopathogenesis involving adaptive immune responses, these mice express human drug‐metabolising enzymes and display human‐specific pharmacokinetic profiles, thereby enabling more accurate prediction of drug metabolism, toxicity and efficacy and bridging the gap between rodent studies and human trials [29, 30]. In this study, mice with high levels of liver chimerism were infected with HEV‐Gt1 and developed stable viral titres that persisted throughout the observation period. By treating the mice with the adenosine nucleoside analogue NITD008, we demonstrate that the model is well‐suited to studying direct antiviral agents, such as RBV and NITD008, against HEV in vivo. NITD008 is a broad‐spectrum chain‐terminating polymerase inhibitor initially developed against flaviviruses and in vitro studies showed that it potently inhibits HEV replicon systems [18, 19]. By treating stably infected humanised mice with NITD008, we demonstrate the very high potency of this class of direct antiviral agents against HEV. Given the substantial reduction in HEV in the liver detected by qPCR, by employing highly sensitive RNA ISH and immunofluorescence staining, the data suggest that direct antiviral drugs against HEV, like NITD008, can substantially contribute to achieving viral clearance even in immunosuppressed patients, when used alone or in combination.

After 2 weeks of treatment, low HEV RNA levels were still detected in the liver, enabling viral rebound after treatment cessation. Moreover, we detected a single nucleotide substitution leading to an amino acid exchange, lysine (K)‐to‐arginine (R) at position 1582 of the C‐terminal end of the functional RNA‐dependent RNA polymerase (RdRp) in one of the two mice that rebounded after treatment cessation. The RdRp was described as adopting a canonical ‘right‐hand’ fold, in which the active site is enclosed by the palm, fingers and thumb domains, with the catalytic motifs distributed across the palm (motifs A–D) [31] and the fingers domain. Motif D is a determinant of nucleotide selection and is involved in the fidelity and speed of these polymerases. Of note, a K to R exchange in the Motif D of the poliovirus RdRp has been reported to modulate the catalytic efficiency of nucleotide incorporation [32]. Although the mutation is not located within the catalytic centre, detecting this variant after NITD008 treatment suggests that reduced incorporation efficiency, combined with increased fidelity, might disfavour incorporation of a chain‐terminating analogue such as NITD008, since this residue appears remarkably conserved across the different members of Hepeviridae. Nevertheless, this subdomain of the HEV polymerase remains poorly characterised and further studies will be needed to assess the consequences of the K1582R variant and determine whether it may impair NITD008 efficacy. As previously reported, treatment‐induced emergence of mutations was not necessarily causally linked to resistance to RBV treatment [9].

RBV, as a broad‐spectrum antiviral, is commonly used in combination with other agents. For example, its combination with interferon‐*α* has been a cornerstone in the treatment of hepatitis C virus (HCV) infections, significantly improving sustained virological response rates compared with monotherapy. Similarly, RBV has been investigated in combination with pegylated interferon for chronic HEV infections, showing improved outcomes in immunocompromised patients [33]. Moreover, combination therapy may also reduce the likelihood of resistance development and shorten treatment duration. These examples underscore the value of combination therapy in managing complex viral diseases. By lowering in vivo infection loads by 5 log10 in only 11 days of treatment, the study demonstrates that RBV combined with low concentrations of a chain‐terminating polymerase inhibitor such as NITD008 (5 mg/Kg, daily) is highly effective in reducing HEV in liver, blood and faeces to nearly undetectable levels in less than 2 weeks.

Although we did not observe any toxic effects in our in vivo experiments with NITD008 during the compound's preclinical evaluation, toxic effects were reported in dogs and rats following prolonged, higher dosing [12], underscoring the need for further development and thorough assessment of this class of compounds. Yet, the strong antiviral potency is encouraging, as potent viral reduction in serum, faeces and liver was achieved within days of treatment. Very recently, Zhang et al. demonstrated a substantial antiviral effect of an alternative guanosine analogue, JNJ‐9117, in vitro and using rat‐HEV in nude rats [10]. Although virus and host differences need to be taken into account, these studies, taken together, reveal that direct antiviral agents, such as nucleoside analogues, exhibit high antiviral potency in the setting of acute or chronic HEV infections. Development of direct antiviral agents is urgently needed, particularly for patients with underlying liver diseases and immunocompromised patients with chronic Hepatitis E, as they often don't respond to or cannot be treated with current drugs like RBV and interferon. However, further preclinical evaluation and screening of additional nucleoside inhibitors, exploration of structure–activity relationships for less toxic derivatives and evaluation of various combination strategies in relevant in vitro and in vivo models of HEV infection are warranted before initiating clinical trials.

## Author Contributions

Initiated and supervised the study: Marc Lütgehetmann, Maura Dandri. Designed experiments: Annika Volmari, Florian Hinte, Marc Lütgehetmann, Maura Dandri. Generated chimeric mice: Tassilo Volz, Lena Allweiss. Performed analyses and generated data: Annika Volmari, Florian Hinte, Tassilo Volz, Junfei Chen, Lena Allweiss. Wrote the manuscript: Annika Volmari, Florian Hinte, Marc Lütgehetmann, Maura Dandri. Corrected the manuscript: Tassilo Volz, Lena Allweiss.

## Funding

MD, ML and LA receive funding from the German Centre for Infection Research (DZIF; TTU Hepatitis 05.820; TTU Hepatitis 05.714; TTU Hepatitis 05.823; TTU Hepatitis 05.726); MD is also supported by the Deutsche Forschungsgemeinschaft (DFG, German Research Foundation)– SFB 1648/1 2024 –512741711 and SFB 1700/1 2025 – 530990199.

## Conflicts of Interest

M. Dandri participates in advisory boards/consultantships and receives funding from Gilead Sciences, Vir. Bio and Aligos.

## Supporting information


**Figure S1:** HEV RNA in serum and faeces during viral rebound following end of treatment in individual mice. HEV RNA in serum (A) and faeces (B) of mice treated with NITD008 for 2 weeks and followed after the end of treatment for a 3 week rebound phase (*n* = 2), compared to untreated control animals (*n* = 2). Control mice are shown in grey and mice receiving NITD008 in blue.

**Figure S2:** Targeted amplicon next generation RNA sequencing of the HEV RpRd following NITD008 treatment. Geneious alignments using samples from an untreated mouse at baseline and at the end of the experiment and two NITD008‐treated mice following rebound, as well as the input virus stock revealed a single nucleotide substitution in one of the two mice after treatment, resulting in an amino acid exchange from lysine (K) to arginine (R) at position 1582 (A). Mean coverage exceeded 10 000‐fold in all five amplified samples and sequences were compared using a detection threshold of 5% for minority variants (B).

**Figure S3:** HEV RNA in serum and faeces following combination treatment in individual mice. HEV RNA in faeces (A) and serum (B) of single mice receiving either RBV or NITD008 monotherapy or a combination of both.

## Data Availability

The data that support the findings of this study are available from the corresponding author upon reasonable request.
